# Factors affecting the success of CT-guided core biopsy of musculoskeletal lesions with a 13-G needle

**DOI:** 10.1007/s00256-023-04477-3

**Published:** 2023-10-18

**Authors:** Khaldun Ghali Gataa, Fatih Inci, Pawel Szaro, Mats Geijer

**Affiliations:** 1 Department of Radiology, Institute of Clinical Sciences, Sahlgrenska Academy, University of Gothenburg, 405 30 Gothenburg, Sweden; 2 Department of Musculoskeletal Radiology, Sahlgrenska University Hospital, Gothenburg, Sweden; 3 Department of Descriptive and Clinical Anatomy, Medical University of Warsaw, Warsaw, Poland; 4 Department of Clinical Sciences, Lund University, Lund, Sweden

**Keywords:** Tomography, X-ray computed, CT-guided, Biopsy, Needle, Diagnostic yield, Bone tumour

## Abstract

**Objective:**

To determine the value of CT-guided bone core biopsy and investigate factors that affect diagnostic yield and biopsy outcome.

**Materials and methods:**

The single-centre retrospective analysis included 447 patients who had CT-guided core biopsy with a 13-G needle (Bonopty®) from January 2016 to December 2021. Histological results or ≥ 6 months of clinical and radiological follow-up served as outcome references. A successful biopsy was classified as “diagnostic” when a definitive diagnosis was made and “adequate” when only the malignant or benign nature of the tumour could be determined. Biopsies were “nondiagnostic” when the nature of the lesion could not be determined. The occult lesions were defined as not seen on CT but visible on other modalities.

**Results:**

In 275 (62%) females and 172 (38%) males, the overall success rate was 85% (383 biopsies), with 314 (70%) diagnostic biopsies and 69 (15%) adequate biopsies. There was no relationship between biopsy success and the localisation of the lesion, length of biopsy material, or number of biopsy attempts. The lesions’ nature had a statistically significant effect on biopsy success with lytic and mixed lesions having the highest success rate. Occult lesions had the lowest success rate.

**Conclusion:**

CT-guided bone core biopsy is an effective method in the workup of musculoskeletal diseases with the highest success rate in lytic and mixed lesions. No apparent relationship was found between biopsy success and biopsy length, number of attempts, or localisation of the lesion.

## Introduction

### Background

Biopsy of musculoskeletal lesions is valuable in the workup of various diseases and malignancies, but biopsies can be challenging procedures with a risk of complications [1]. An adequate biopsy is essential to determine the nature of lesions to guide medical management and treatment decisions. Although modern radiological imaging techniques have a good diagnostic value in differential diagnosis, a histopathological examination is usually necessary to make a final diagnosis and plan further treatment [2]. CT-guided percutaneous needle biopsy of musculoskeletal lesions is a well-described technique for determining the nature of indeterminate lesions, and the high diagnostic accuracy and safety of the procedure are well-documented [3].

Percutaneous needle biopsy has several advantages over open biopsy. It is less invasive, thus minimising infection risk and wound-related complications and can be performed in an outpatient clinic or day hospital since general anaesthesia is rarely necessary. These features lead to a safer profile compared to open biopsy, reduced costs, shorter procedural length, shorter hospital stays, and fewer complications. CT-guided percutaneous needle biopsy is generally considered safe and effective, and there is a low risk of complications such as bleeding, infection, nerve damage, or damage to nearby organs [2, 3]. Moreover, CT guidance provides a better 3D orientation and handling capacity of the area of interest during biopsy [4].

### Rationale

Many biopsy-related factors may theoretically affect the success of musculoskeletal lesion biopsy, including the location of the lesion, relationship to vital and sensitive structures, nature of the lesion, biopsy equipment, and sample size. These factors could impact diagnostic accuracy and success and should be considered [5]. Multiple biopsy attempts and longer specimens have been reported to result in a higher proportion of successful biopsies [6]; however, other reports have not managed to replicate this finding [7]. The purpose of the current study was to assess the diagnostic accuracy of CT-guided core biopsy of musculoskeletal lesions and factors that affect biopsy success in a single tertiary institution.

## Materials and methods

### Study design

This is a retrospective analysis of CT-guided biopsies of musculoskeletal lesions.

Ethical approval for the current study was obtained from the National Ethical Review Authority and the requirement for informed consent was waived (2021-00466, 2022-01968-02).

### Patients, biopsy procedure, and specimen assessment

**Fig. 1:**
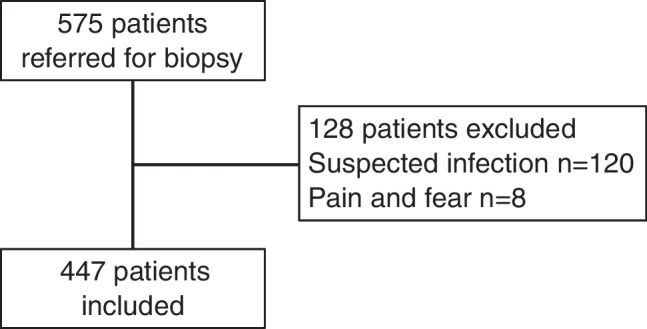
Inclusion of patients in the study

In this retrospective analysis, data were collected for all patients that underwent CT-guided core biopsy of musculoskeletal lesions between 1 January 2016 and 31 December 2021 at the Musculoskeletal Radiology section of our university hospital for indeterminate lesions where malignancy could not be excluded. Biopsies were excluded from this study if the main referral reason was suspicion of infection or in the case of inability to perform the biopsy due to severe pain, panic attacks, or anxiety. Fifty-three biopsies for which the length of biopsy material was not recorded were excluded from the analysis of the relationship between biopsy success and biopsy length. After exclusions, 447 patients with suspected malignancy were included (Fig. 1). Patients for whom only the malignant or benign nature of the tumour could be determined were followed up to confirm the accuracy of biopsy results using the results from histological analyses and follow-ups including both clinical and radiological data of ≥ 6 months as outcome references.

All procedures were performed using a Somatom Definition (Siemens Healthineers, Erlangen, Germany) or Aquilion Prime (Canon Medical Systems Corporation, Tochigi, Japan) CT scanner. A 13-G Bonopty Biopsy Set (Aprio Med AB, Uppsala, Sweden) was used for all biopsies. The CT machines used in the study had the capability for three-plane reconstruction with navigation to the target lesion. All procedures were done by one of four musculoskeletal radiologists with 3–7 years of experience in musculoskeletal and interventional radiology. Biopsies were evaluated at the Pathology Department of our university hospital with no strict definitions related to the assessment of biopsy effectiveness.

### Definitions of variables

A CT-guided core biopsy was considered “diagnostic” when a definitive histological diagnosis could be made and “adequate” when only the malignant or benign nature of the tumour could be determined. Biopsy success was defined as the sum of diagnostic and adequate biopsies. A biopsy was considered “nondiagnostic” when the nature of the lesion could not be identified. Lesions were considered occult when they could not be visualised on CT examination but had previously been detected on positron emission tomography or magnetic resonance imaging. Occult lesions were targeted using anatomical landmarks to come as close as possible to the lesions. We used the term “soft tissue component” to refer to the soft tissue extension arising from a lesion originally located in the bone. The biopsy of the soft tissue component was performed through the same access point, without altering the position of the guiding needle and during the same procedure as the bone biopsy.

### Statistics

Fisher-Freeman-Halton’s exact test was used to assess the relationships between biopsy success and the number of biopsy attempts, the localisation of the lesion, and lesion characteristics. Cochran-Mantel-Haenszel’s test (linear-by-linear association) was used to evaluate the relationship between biopsy success and the length of biopsy material. The relationship between success of biopsy and interventionist who took biopsy was analysed statistically with Pearson’s chi-square test. A *p*-value of ≤ 0.05 was considered statistically significant.

## Results

Of the 447 biopsies, 275 (62%) were performed on female and 172 (38%) on male patients. The age range was 5 to 89 years, with a mean age of 61.9 ± 16.8 at the procedure time. Nine biopsies were conducted on individuals under 18 years of age where diagnosis could be made in 8 of them. According to the CT characteristics, 181 (41%) lesions were osteolytic, 146 (33%) sclerotic, and 60 (13%) mixed type, and 36 (8%) lesions had a soft tissue component that was biopsied with the bony component. Twenty-four (5%) lesions were classified as occult on CT. CT-guided bone core biopsy was most commonly performed under local anaesthesia (*n* = 354, 79%) or local anaesthesia combined with an intravenous sedative agent (*n* = 65, 14%). Twenty-one procedures (5%) were done under general anaesthesia, and in 7 cases (2%), the type of anaesthesia was not recorded.

Biopsies were diagnostic in 314 (70%) cases and adequate in 69 (15%), with a total success rate of 85% (*n* = 383). Nondiagnostic biopsies were recorded in 64 cases (15%). Nine percent of the biopsies were primary musculoskeletal tumours (n = 42), while 60% (n = 267) were metastases, and the rest resulted in other diagnoses of non-tumorous origin such as sarcoidosis, hyperplasia, or normal trabecular bone. Breast cancer was the most common metastasis (43%), while oesophageal cancer was the least common (1%). Chondroma was the most common primary musculoskeletal tumour, representing 12% of all primary tumours. The spine was the most commonly biopsied part of the skeleton, accounting for 49% of all biopsies.

**Fig. 2:**
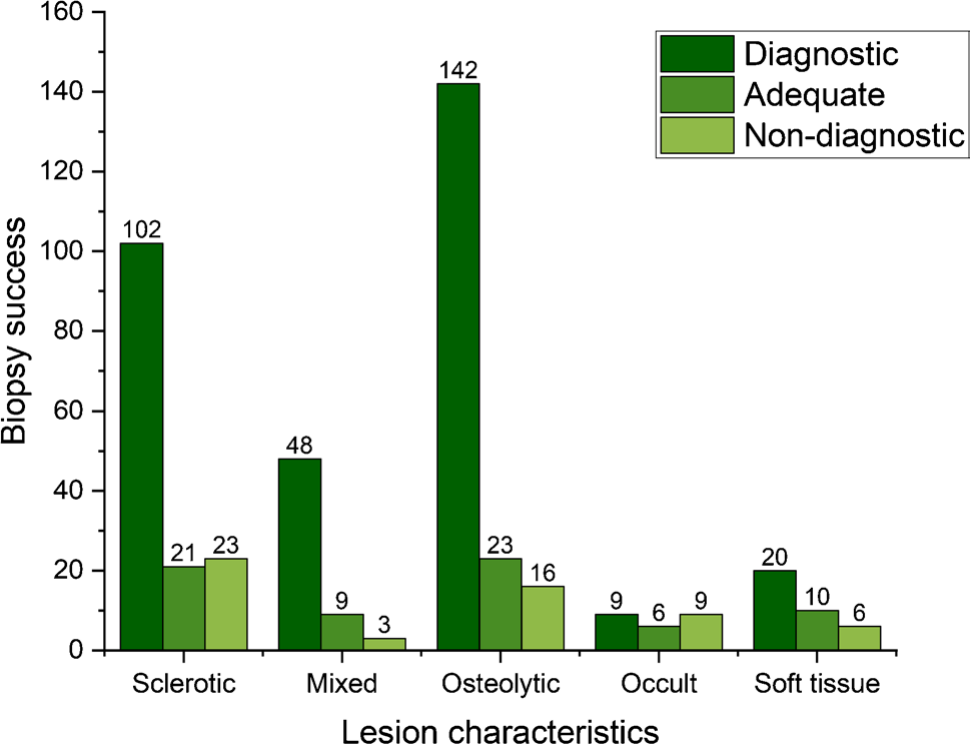
Number of successful biopsies in relation to lesion character

**Fig. 3:**
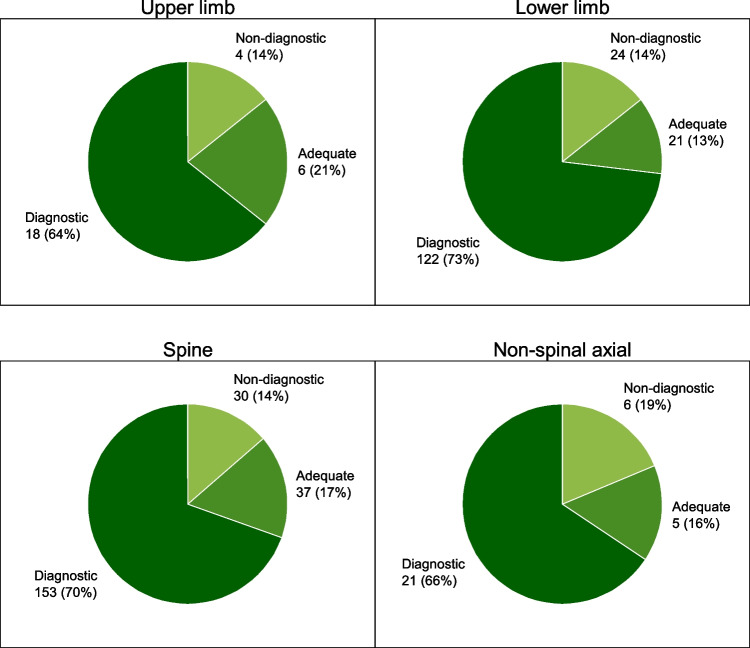
Success of biopsy in relation to localisation

There was a strong relationship between biopsy success and lesion characteristics with the highest success rate for osteolytic and mixed lesions and the lowest for occult lesions (*p* = 0.001; Table 1; Fig. 2). For the variables of interest, there was no statistically significant difference between either the number of biopsy attempts (*p* = 0.243) or the length of biopsy material (*p* = 0.769). No statistically significant relationship was found between sex and the localisation of the lesion or between biopsy success and the localisation of the lesion (*p* = 0.636; Fig. 3). No statistically significant relationship was found between biopsy success and the type of anaesthesia.

**Table 1:** Success rate of biopsies in relation to different studied variables

Variables		Successful	Diagnostic	Adequate	Nondiagnostic
Localisation	Upper limb	24 (5.3%)	18 (4%)	6 (1.3%)	4 (0.9%)
	Lower limb	143 (32.0%)	122 (27.3%)	21 (4.7%)	24 (5.4%)
	Spine	190 (42.5%)	153 (34.2)	37 (8.3%)	30 (6.7%)
	Axial skeleton excluding the spine	26 (5.8%)	21 (4.7%)	5 (1.1%)	6 (1.3%)
Character	Sclerotic	123 (27.5%)	102 (22.8%)	21 (4.7%)	23 (5.1%)
	Mixed	57 (12.7%)	48 (10.7%)	9 (2.0%)	3 (0.7%)
	Osteolytic	165 (36.9%)	142 (31.8%)	23 (5.1%)	16 (3.6%)
	Occult	15 (3.3%)	9 (2.0%)	6 (1.3%)	9 (2.0%)
	Soft tissue	30 (6.7%)	20 (4.5%)	10 (2.2%)	6 (1.3%)
Number of biopsy attempts	1	190 (42.5%)	149 (33.3%)	41 (9.2%)	39 (8.7%)
	2	113 (25.3%)	97 (21.7%)	16 (3.6%)	15 (3.4%)
	3	47 (10.5%)	40 (8.9%)	7 (1.6%)	4 (0.9%)
	4 and more	33 (7.4%)	28 (6.3%)	5 (1.1%)	6 (1.3%)
Length of biopsy	2–10 mm	92 (23.4%)	72 (18.3%)	20 (5.1%)	10 (2.5%)
	11–18 mm	93 (23.6%)	72 (18.3%)	21 (5.3%)	10 (2.5%)
	19–25 mm	83 (21.1%)	70 (17.8%)	13 (3.3%)	11 (2.8%)
	26–109 mm	85 (21.6%)	74 (18.8%)	11 (2.8%)	10 (2.5%)
Type of anaesthesia	Local anaesthesia	305 (68.3%)	247 (55.3%)	58 (13.0%)	49 (11.0%)
	Local anaesthesia + intravenous sedation	56 (12.5%)	47 (10.5%)	9 (2.0%)	9 (2.0%)
	General anaesthesia	16 (3.6%)	15 (3.4%)	1 (0.2%)	5 (1.1%)
	Not recorded	6 (1.3%)	5 (1.1%)	1 (0.2%)	1 (0.2%)
Interventionist	Interventionist-1	81 (18.1%)	67 (15.0%)	14 (3.1%)	10 (2.2%)
	Interventionist-2	85 (19.1%)	70 (15.7%)	15 (3.4%)	13 (2.9%)
	Interventionist-3	95 (21.3%)	83 (18.6%)	12 (2.7%)	14 (3.1%)
	Interventionist-4	125 (28.0%)	106 (23.7%)	19 (4.3%)	24 (5.4%)

No influence of the operator experience on biopsy success was found in this study (*p* = 0.87) (Table 1).

## Discussion

The present study demonstrated that lesion characteristics have a strong relationship with the success of musculoskeletal biopsies. Even though the efficacy of CT-guided biopsy has been widely studied, ranging between 64 and 97% across published reports, there is little published evidence on the factors that affect the success of musculoskeletal biopsies [7–10]. Factors that may affect the diagnostic yield of biopsies include the nature of lesions, the length of biopsy material, the number of biopsy attempts, the size of the biopsy needle, and the size of the lesion [6, 7, 11].

In this study, the overall success rate of biopsies was 85%, which is in keeping with results from other studies showing that CT-guided musculoskeletal biopsy is an effective procedure that plays a crucial role in diagnosing suspected musculoskeletal lesions [12–15]. Metastases were the most frequent finding in biopsies, with breast cancer as the most common type (43%), while only 9% of biopsies showed primary musculoskeletal tumours. These results reflect the epidemiology of malignant diseases and patterns of metastasis of primary tumours [14, 16]. The spine was the most common biopsy site in this study (49%). This finding is in alignment with previous studies, which identified the spine as the most common site for metastasis due to the vascular red marrow and communication of valveless deep torso veins with the vertebral venous plexus [14, 17].

No statistically significant relationship was found between the success of biopsy and biopsy localisation, which confirms findings from other studies [1]. This suggests that biopsy success is primarily driven by the nature of lesions. Yet, our findings do not diminish the role of biopsy-related factors, such as the localisation of the lesion, which introduce procedural challenges regarding how to target the lesion and avoid complications. Thanks to the capability of utilising multiplanar reconstruction (MPR) and 3D imaging, precise navigation of the biopsy needle is more achievable. If needed, an accurate correlation of the needle’s position in three planes is possible, greatly facilitating spatial understanding of its location in relation to the target lesion. The results from the current study differ from those of Hau et al., where accuracy was lower for spinal biopsies than for pelvic biopsies, which was thought to be related to lesion size that is often larger in pelvic lesions [18]. Accurately positioning, advancing, and manipulating instruments in relation to the target lesion define handling capacity in CT guidance. This entails manipulating the biopsy needle with steadiness, control, and precision and is a crucial factor in the process. Neither the length of biopsies nor the number of biopsy attempts per procedure affected biopsy success in the current study. This is in line with a systematic review by Michalopoulos et al. which found no relationship between success and the gauge of the needle [1]. However, in contrast with the results reported by Wu et al. [11], the current study has not found a statistically significant relation between biopsy success and length of biopsy. In the current study, all biopsies were done with 13-G needles, while Wu et al. used 14-, 15-, 16-, and 18-G needles [11]. A larger bone core needle diameter gives a larger volume of biopsy material even for the same needle length. For example, a 10-mm-long specimen obtained with an 11-G needle has a size of 44.8 mm^3^ compared to 14.78 mm^3^ for a 15-G needle, i.e. 303% higher volume [8]. In a report by Chang et al., the success of biopsies taken with 11-G needles was 84.6% compared to 63.6% with smaller diameter needles [8]. One could advocate for the use of needles with larger diameters, which may decrease the number of biopsy attempts needed. It is believed that it would give better results even for a smaller number of biopsy attempts due to the larger volume of sample obtained. The discussion regarding the optimal biopsy needle for obtaining tissue samples from the skeleton is complex and stems from the fact that authors define success based on the possibility of achieving a histological diagnosis. In our study, we concentrate on a specific needle thickness, 13G, and illustrate that the length of the biopsy has little impact on the ability to establish a diagnosis. Even variations among the four operators who conducted the biopsies do not yield statistically significant differences. Furthermore, we identified a strong relationship between biopsy success and lesion characteristics, with the highest success rate observed for osteolytic and mixed lesions and the lowest for occult lesions. We believe that the 13G thickness represents an optimal compromise, considering the potential complications associated with using a thicker needle and the risk of nondiagnostic biopsies with a thinner needle. However, to comprehensively address the question of comparing different needles, additional studies utilising varying thicknesses should be undertaken. The use of a larger diameter needle in the current study, 13-G, resulted in a high success rate, but this needs to be investigated further. Future studies should compare different needle sizes to determine which size provides the best diagnostic yield for biopsies of musculoskeletal lesions.

The highest success rate for biopsies in this study was found in lytic lesions followed by mixed lesions. In contrast, a systematic review by Michalopoulos et al. [1] showed no relationship between the results of biopsies of lytic, sclerotic, or mixed lesions, while earlier retrospective studies reported that sclerotic lesions had a lower success rate compared to lytic lesions [7, 11]. In the current study, the biopsy success rate was lowest for occult lesions, 62% (37% diagnostic and 25% adequate biopsies), which is expected considering the greater difficulty in targeting invisible lesions that are typically smaller and have lower cellular density than visible lesions [12]. The success of targeting occult lesions in this study was higher than previously reported, e.g. 37.1% by Wonsuk et al. [19]. Thus, our work advocates the use of CT-guided bone core biopsy in the workup of invisible lesions but at the same time highlights the need to develop more adequate techniques to increase the diagnostic yield [19]. Future studies should focus on developing new techniques that can improve the diagnostic yield for occult lesions.

There are some limitations to the current study. As with most studies on image-guided bone core biopsy, the current study has a retrospective design and is impacted by typical limitations for this type of study, such as difficulties in the retrieval of information like the length of some biopsies and the number of biopsy attempts [8, 14, 18–27]. Further, the study only included patients from a single university hospital and used only one type of biopsy set which may limit its generalisability to other populations or settings.

In conclusion, the nature of the musculoskeletal lesions was the main factor affecting the results of CT-guided core biopsy using a 13-G needle. No statistically significant influence was found for the localisation of the lesion, number of biopsy attempts per procedure, type of anaesthesia, or length of biopsy material. There is a need to develop better techniques to biopsy occult lesions to improve success rates.

This study was done on a relatively large number of patients with the use of one type of relatively large needle that minimises spreading of factors that may affect success of biopsies. The most significant result of our study is that using a 13G needle for CT-guided skeletal biopsies may help reduce the need for unnecessary additional punctures.

## Data Availability

The datasets analyzed during the current study are not publicly available due to the Swedish legislation (the Personal Data Act), but a limited and fully anonymized data set that supports the main analyses is available from the corresponding author on request.
